# Air pollution, temperature, and HbA1c levels among children in Mexico City, Mexico

**DOI:** 10.1016/j.ecoenv.2025.119424

**Published:** 2025-11-19

**Authors:** Jeanne Wu, Pablo Knobel, Mike Z. He, Itai Kloog, Allan C. Just, Ivàn Gutiérrez-Avila, Elena Colicino, Martha M. Téllez-Rojo, María Luisa Pizano-Zárate, Marcela Tamayo-Ortiz, Alejandra Cantoral, Diana C. Soria-Contreras, Andrea A. Baccarelli, Robert O. Wright, Maayan Yitshak Sade

**Affiliations:** a Department of Environmental Medicine, Icahn School of Medicine at Mount Sinai, New York, United States; b Department of Geography and Environmental Development, Ben-Gurion University of the Negev, Beer Sheva, Israel; c Department of Epidemiology, Brown University School of Public Health, Providence, RI, United States; d Center for Nutrition and Health Research, National Institute of Public Health, Cuernavaca, Morelos, Mexico; e Nutrition and Bioprogramming Coordination, National Institute of Perinatology, Mexico, Mexico; f UMF 4, South Delegationof the Federal District, Mexican Social Security Institute (IMSS), Mexico, Mexico; g Occupational Health Research Unit, Mexican Social Security Institute (IMSS), Mexico, Mexico; h Department of Health, Universidad Ibeoamericana, Mexico, Mexico; i Diabetes Research Center, Massachusetts General Hospital, MA, United States; j Department of Environmental Health Sciences, Columbia University Mailman School of Public Health, New York, United States

**Keywords:** Glycemic control, HbA1c, Children, Mexico, Air pollution, Temperature

## Abstract

Glycated hemoglobin (HbA1c), a strong indicator of diabetes, is affected by air pollution and temperature exposures. However, studies examining the association between HbA1c, temperature, fine particulate matter (PM_2.5_), and nitrogen dioxide (NO_2_) among children are scarce, especially looking at all exposures simultaneously. We investigated the associations between these exposures and HbA1c among children, known to be a highly susceptible group. We included children enrolled in the Programming Research in Obesity, Growth, Environment, and Social Stressors (PROGRESS) cohort based in Mexico City (2013–2019). We obtained exposures from spatiotemporal models. HbA1c levels were measured at 4–5 years, 6–7 years, and 8–11 years post-birth. We used multivariable linear mixed-effects models to assess the simultaneous associations of three-month averages of PM_2.5_, temperature, and NO_2_ exposures with natural log-transformed HbA1c levels. We fitted stratified models by sex and age group. We included 1186 HbA1c measurements from 503 children. In multi-exposures models 1-μg/m^3^ increase in PM_2.5_ was associated with 0.311 % (95 % CI:0.159, 0.464) relative increase in HbA1c and 1-degree Celsius increase in average temperature was associated with a −0.626 % (95 % CI: −0.977, −0.274) relative decrease. We did not find an association with a 1-ppb increase in NO_2_ (−0.010 %. 95 % CI: −0.126, 0.106). The stratified analysis found slightly different associations by sex and age. This study adds evidence of HbA1c increases associated with lower temperatures and higher PM_2.5_ exposure in an area with high air pollution and moderate temperature fluctuation. These glycemic changes may translate into increased diabetes risk in later life.

## 1. Introduction

Glycated hemoglobin (HbA1c) reflects average blood glucose levels over the preceding two to three months. Unlike blood glucose measurements, HbA1c is not influenced by postprandial fluctuations, making it a stable indicator of long-term glycemic control (Tian et al., 2022; Zhang et al., 2010). Elevated HbA1c levels have been linked to increased cardiometabolic risk (Lee et al., 2013), all-cause mortality (Matsushita et al., 2010), and the development of youth-onset type two diabetes (Zhang et al., 2010).

Studies show that environmental exposures, including air pollution and temperature, are associated with increased cardiometabolic risk in adults (Khoshhali et al., 2021) and children (Liu et al., 2024). A 2022 meta-analysis found that independent increases in PM_2.5_ and NO_2_ were associated with increases in HbA1c levels in adults (Tian et al., 2022). Ambient temperatures also have been suggested to be associated with glycemic status, but the evidence is limited and inconsistent (Khoshhali et al., 2021; Alghamdi et al., 2021; Tien et al., 2016). Several gaps still need to be addressed in the current evidence base. First, most studies focus on single exposures. While a few studies incorporate PM_2.5,_ NO_2_, and temperature in their analyses, most treat temperature as a confounder (Yitshak Sade et al., 2016; He et al., 2022) and analyze the associations between air pollutants and HbA1c separately using single exposure models (Riant et al., 2018; Hwang et al., 2020). PM_2.5,_ NO_2_, and temperature – as many other environmental exposures – do not present in isolation, especially in dense cities, and a systematic review found cumulative and potentially synergistic health impacts of air pollution and heat (Anenberg et al., 2020).

Second, most studies of air pollution and glycemic outcomes focus on the general adult population. The limited evidence among children suggests an association between HbA1c and PM_2.5_ (Moody et al., 2019), but not with NO_2_ (Tian et al., 2022). Focusing on children is vital because metabolic health impacts of environmental exposures during childhood can translate into cardiovascular impacts in adulthood (Guo et al., 2024). Moreover, childhood might be a critical window of susceptibility to atmospheric environmental exposures, as children inhale more air per unit time, have a smaller lung surface area, and have less mature pulmonary function compared to adults (Makri and Stilianakis, 2008). This vulnerability is especially relevant considering the increasing trends of cardiovascular risk factors, including obesity and diabetes, in both children and adolescents (Daniels, 2001; Rodriguez et al., 2006). Lastly, there might be different levels of vulnerability among children based on sex or developmental stage (Wang et al., 2020). Children at different developmental stages may exhibit varying vulnerabilities to environmental exposures, as biological and social factors—such as hormonal changes, lung development, and activity patterns (Wang et al., 2020)—can modulate their susceptibility. Further, limited evidence supports sex differences in susceptibility to environmental atmospheric exposures (Silveyra et al., 2021).

In this study, we investigate the concurrent associations of HbA1c with PM_2.5_, NO_2_, and ambient temperature among children enrolled in the Programming Research in Obesity, Growth, Environment, and Social Stressors (PROGRESS) cohort in Mexico City (Barraza-Villarreal et al., 2008). Moreover, we investigate the differences of the association by sex and age.

## 2. Methods

### 2.1. Study population and outcome

The study population consists of the children recruited in the PROGRESS cohort of Mexico City, a metropolitan area noted as having some of the worst air quality in the world and the densest population in all of Mexico (Gouveia et al., 2021). The topography around Mexico City leads to poor ventilation and light winds, often trapping air pollutants within the region (Bravo-Alvarez and Torres-Jardón, 2002). The temperature range of Mexico City is rather mild and seasonal changes are typically categorized by weather and humidity with a cold-dry season from November to February, a warm season with low humidity in March to April, and a rainy season from May to October (Miquelajauregui et al., 2024).

A total of 1054 mothers were recruited during their second trimester of pregnancy and followed along with their children born between July 2007 and February 2011. The mothers were considered eligible to enter the cohort if they were over the age of 18, less than 20 weeks pregnant, free of heart or kidney disease, did not use steroids or anti-epilepsy drugs, did not consume alcohol daily, had access to a telephone, and planned on residing in Mexico City for the next three years. A total of 503 children from the cohort were included in the analytical dataset. The children’s measures of HbA1c were obtained at 4–5 years, 6–7 years, and 8–11 years old, between 2013 and 2019. In addition to blood measurements, data collected from the PROGRESS cohort mothers include age, marital status, smoking status, and education. More information on the recruitment process and data gathered from the PROGRESS cohort has been previously published (Renzetti et al., 2017). This cohort study was approved by the Institutional Review Boards at the Mexico National Institute of Public Health (CI-1873-20092023) and the Icahn School of Medicine at Mount Sinai (STUDY-12-00751A). Participants were excluded from our analytical dataset if their residential histories (20.8 %) or covariate information (5 %) was missing.

### 2.2. Exposure assessments

We obtained daily 1-km^2^ predictions of PM_2.5_, NO_2_, and ambient temperature over Mexico City using hybrid satellite modeling approaches. For PM_2.5_ we used both extreme gradient boosting and inverse-distance weighting techniques that incorporate aerosol optical depth, meteorology, and land-use variables to generate daily predictions at 1-km resolutions. For NO_2_ we applied a unique hybrid ensemble model which incorporates machine-learning (XGBOOST, RF), geo-statistics and remote sensing approaches. Daily temperature data were obtained using a calibrated model that integrated satellite-derived surface temperature readings with ground-based air temperature measurements, employing land use regression techniques for adjustment. All models were evaluated using cross-validation methods and demonstrated good performances. More information on the spatiotemporal models can be found in corresponding literature (Gutiérrez-Avila et al., 2021, 2022; He et al., 2023).

The home latitudes and longitudes of PROGRESS participants, derived from their geocoded residential histories to account for potential residential mobility over time, were matched to the corresponding 1-km^2^ grid cells of the satellite-based model predictions. PM_2.5_, NO_2_, and ambient temperature levels were determined with three-month averages prior to each HbA1c draw. Three-month averages were selected as the exposure window of interest based on previous evidence from this cohort as having the more substantial associations (He et al., 2022).

### 2.3. Statistical analysis

We used linear mixed-effects models to investigate the associations between the exposures (i.e. NO_2_, PM_2.5_, ambient temperature) and HbA1c among children. Given the highly skewed distribution of the outcome, we natural-log-transformed the HbA1c values. First, we investigated the association between three-month averages of the exposures and HbA1c in single-exposure models. To assess the linearity of the associations, we fitted each exposure using penalized splines. After visual inspection, all associations were deemed linear or approximately linear (Figure S1). We added a random intercept for each participant to account for multiple participant measurements. We adjusted the models for the child’s age at the visit, the season of the visit (November-February, March-April, or May-October), and their mothers’ smoking status, marital status, and education level. We added a penalized spline for the year of testing to account for the potential time trend. All coefficients were transformed back to original units and presented as relative percentage changes in the HbA1c levels with 95 % confidence intervals associated with each unit increase in the exposures.

Subsequently, we used multivariable linear mixed-effects models to assess the simultaneous associations of PM_2.5_, NO_2_, and ambient temperature, averaged over three months before the visit, with natural log-transformed HbA1c levels among children. The multivariable model was adjusted for the same set of confounders as the single-exposure models. We used Pearson’s tests to evaluate the correlations between the exposures and assure no collinearity issues in the multivariable model.

As a secondary analysis and to investigate the potential differences by sex and age group, we repeated our multi-exposure model stratifying by sex (male and female) and age group (4–5 years, 6–7 years, and 8–11 years). These analyses were added to capture potential vulnerabilities among sexes, and in different stages of growth and development. Lastly, to evaluate the potential interactions between the exposures we repeated the multi-exposure models for each exposure pair (e.g. PM_2.5_ and NO_2_,).

As sensitivity analysis to address potential selection bias related to the exclusion of children with missing covariate data, we repeated the analysis using stabilized inverse probability weights. We used logistic regression to estimate the probability of having complete HbA1c information, including participants’ age and sex along with mothers’ marital status, alcohol use, and education level, as well as visit number, year, and month – as predictors. Additionally, evidence values (E-values) were estimated to account for how robust our estimates are to unmeasured confounders. The E-value is estimated on the ratio scale, and provides an estimate for the strength of the association an unmeasured confounder must have with both the exposure and the outcome to eliminate the observed exposure-outcome association (Chung and Chung, 2023). Finally, we repeated our analysis using six-month averages instead of three months. Statistical analyses were conducted using R Statistical Software, version 4.4.2. (Foundation for Statistical Computing, Vienna, Austria).

## 3. Results

We included 1196 HbA1c measurements of 503 children with ages ranging from 4 to 11 years (336 in ages 4–5 years, 399 in ages 6–7 years, and 461 in 8–11 years). Hb1ac was measured at least twice in each of the children during the 7 years of follow-up (2013–2019). 51.2 % of participants were females. Among the mothers, 81.4 % were married or had a lifetime partner, 61.7 % reported ever smoking, and 42.9 % had a technical post-middle/high school or high school education (Table 1). Descriptive statistics of the stratified population by sex and age group are shown in Table S1. We observed a higher proportion of follow-up visits in the warm season (May – Oct) among children 8–11 years. This age group experienced lower pollutants exposure and higher temperature exposure compared to earlier visits. The exposure pollutants and temperature exposure levels were similar between sexes. Maternal smoking was more prevalent among females, and a higher proportion of females arrived in the spring (March – April), compared to males. Other population characteristics did not differ by age or sex (Table S1).

**Fig. 1.**
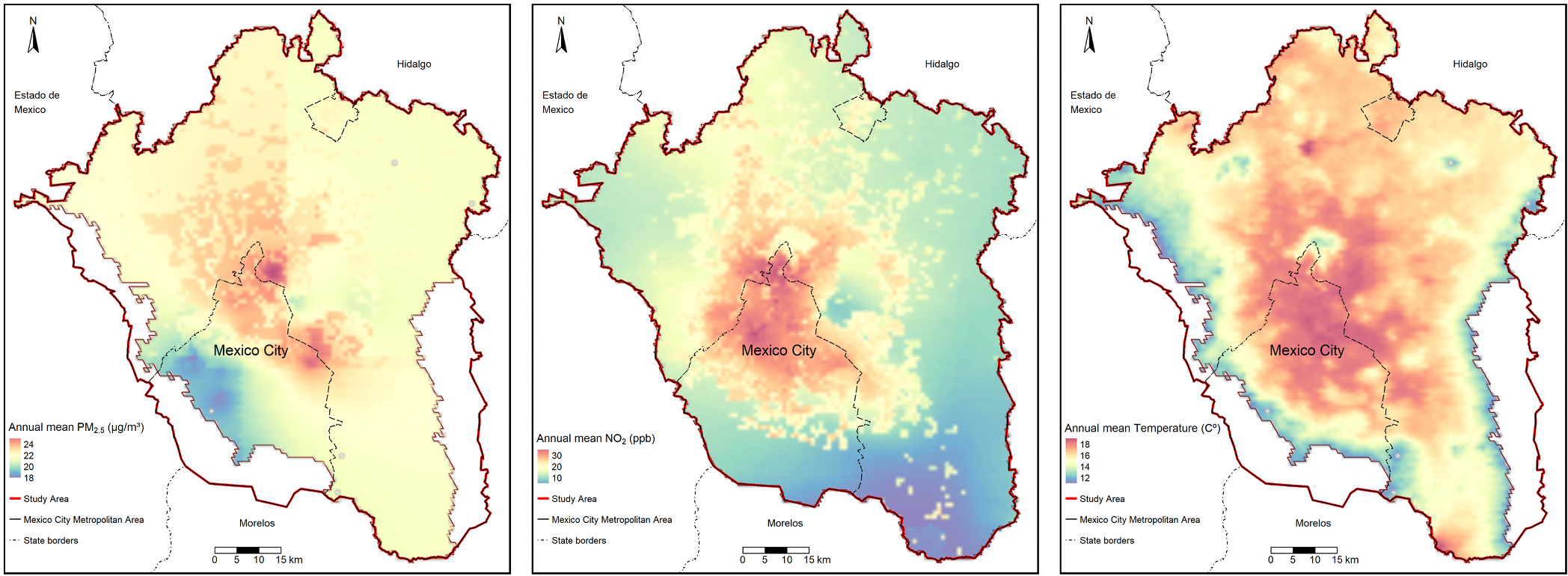
Maps of the average annual mean PM_2.5_, NO_2_, and average temperature for 2015 in the Mexico City Metropolitan Area.

The mean value and standard deviation of the 3-month average exposures were: 20.72 (4.22) μg/m^3^ for PM_2.5,_ 28.87 (6.16) ppb for NO_2,_ and 15.67 (1.97) °C for temperature. Fig. 1 shows the spatial variability in the exposures, with higher levels in the center of Mexico City and lower levels on the outskirts of the study area. It also highlights the differential spatial distribution among the exposures. The correlation between the exposures was low to moderate, with the highest correlation (r = 0.56) between PM_2.5_ and NO_2_ (Figure S2).

In the multi exposure models assessing the associations with 3-month exposure averages, we found a 1 μg/m^3^ increase in PM_2.5_ associated with a 0.311 % (95 % CI: 0.159, 0.464) relative increase in HbA1c and a 1-degree increase in average temperature associated with a −0.626 % (95 % CI: −0.977, −0.274) relative decrease. We did not find an association with a 1 ppb increase in NO_2_ (−0.010 %. 95 % CI: −0.126, 0.091). The results of the single exposure models were very similar. As a sensitivity analysis, we repeated the multivariable model using inverse probability weights to address potential selection bias from missing covariate data. The effect estimates from the weighted model were highly similar to those from the non-weighted model (Table 2). Sensitivity analysis using six-month averages of the exposures also had similar effect estimates (Table S3). We found no evidence for interactions between the exposures. Interaction p-values were 0.332 for PM_2.5_ and temperature, 0.550 for NO_2_ and temperature, and 0.263 for PM2.5 and NO2.

**Fig. 2.**
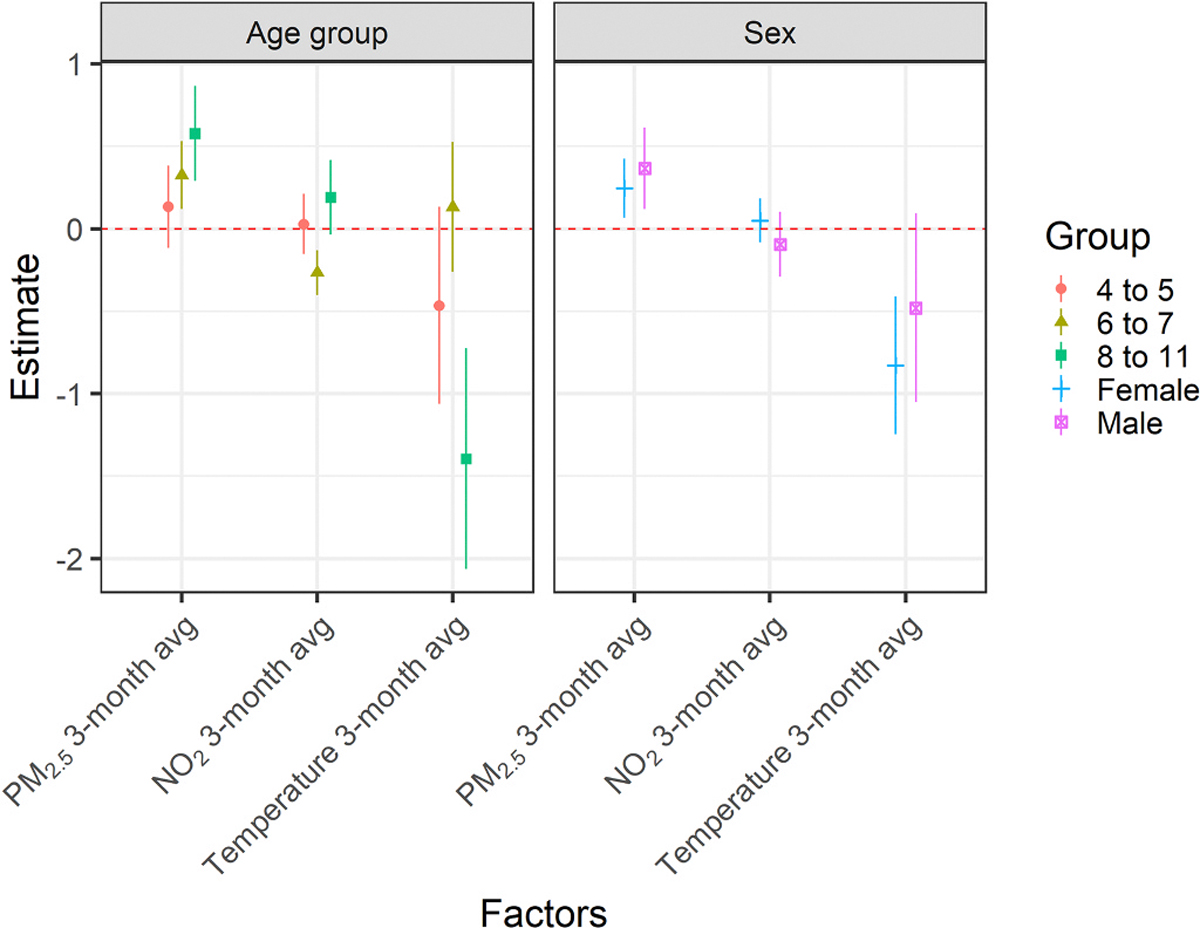
The associations between PM_2.5_, NO_2_, ambient temperature, and HbA1c levels stratified by sex and age group. Results were obtained from multi-exposure models including all three air exposures in a single model while adjusting for the children’s age, season, and year of their clinical visit, as well as their mothers’ smoking status, marital status, and education level. Relative percent changes are calculated for each unit change of exposure.

The association with temperature was more pronounced among females (−0.829 %. 95 % CI: − 1.246, −0.410) compared to males (−0.480 %. 95 % CI: − 1.050, 0.095). The association between PM_2.5_ and HbA1c increased with age. One μg/m^3^ increase in three months average PM_2.5_ exposure was associated with a 0.132 % relative increase in HbA1c (−0.116 %, 0.382 %) among the 4 and 5 age group, 0.325 % relative increase (0.120 %, 0.531 %) among the 6 and 7 age group, and 0.577 % relative increase (0.289 %, 0.866 %) among the oldest age group. The association with temperature also differed by age, with stronger protective effects of hotter temperatures among the youngest (−0.466 %. 95 % CI − 1.062 %, 0.134 %) and oldest (−1.396 %. 95 % CI − 2.062 %, −0.726 %) age groups (Fig. 2, Table S2).

As a sensitivity analysis, we calculated the E-values for the associations with PM_2.5_ and NO_2_. We found that an unmeasured confounder will need to have unlikely strong associations with the outcome and the exposures to eliminate the observed associations with these two exposures. Specifically, a risk ratio of 2.58 to eliminate the association with PM_2.5_, and a risk ratio of 0.23 to eliminate the association with temperature (Table S4).

## 4. Discussion

Novel spatiotemporal models can now estimate ambient environmental data at a 1-km^2^ resolution of which previously were only observed at a 10-km^2^ resolution. We analyzed the simultaneous associations of residential ambient PM_2.5_, NO_2_, and temperature with HbA1c levels among children in the PROGRESS cohort of Mexico City. Higher PM_2.5_ exposure and lower temperature exposure were associated with higher HbA1c levels, while no association was observed with NO_2_. Although it may be difficult to translate HbA1c changes among children surveyed in a four year time span to cardiovascular risk in the future, elevated HbA1c in children is significantly correlated with diabetes in later life (Guo et al., 2024). Among people with diabetes, the most prevalent cause of mortality is cardiovascular disease (Almourani et al., 2019), thus we emphasize the importance of understanding environmental exposures and their impacts to HbA1c.

We found a stronger association between temperature, PM_2.5_, and HbA1c levels in older children. Studies show that as children age their HbA1c levels increase, potentially leading to greater HbA1c variability and increased fluctuations in response to external exposure (Hovestadt et al., 2022; Jansen et al., 2015). The larger effects among older children may also be due to differences in leisure activities or the transition from primary to middle school, both of which can influence exposure patterns. Additionally, associations seen in older children may reflect the cumulative impact of prolonged environmental exposures.^31^Interestingly, we also observed a protective association with NO_2_ among children in the middle age group. However, this finding may be spurious and potentially attributable to residual confounding or unique exposure patterns within this age group. Behavioral, activity-related, social, and exposure pattern differences may also explain the sex differences observed in our study, (Silveyra et al., 2021) which showed a stronger association with temperature among females. Finally, both age and sex differences may be related to biological factors and developmental stages that influence children’s vulnerability to the effects of air pollution.

In our study, exposure to higher concentrations of PM_2.5_ was associated with increased HbA1c levels in children. While these findings on PM_2.5_ and HbA1c levels in children are novel, they align with existing evidence of associations with glucose in children, showing increases in PM_2.5_ associated with increases in fasting glucose levels (Cai et al., 2019). Our findings of no associations between NO_2_ and HbA1c are also consistent with the existing limited literature: a study focusing on children and young adults in Germany found no association between NO_2_ and HbA1c (Tamayo et al., 2016). These overall results are consistent with a systematic review assessing the associations of air pollution with HbA1c in the general adult population, which found consistent associations with PM_2.5_ and less robust associations with NO_2_(Tian et al., 2022). Similarly, the analysis on the mothers of the PROGRESS cohort found increases PM_2.5_ associated with increases in HbA1c levels (Makri and Stilianakis, 2008).

Although the biological mechanisms explaining the links between air pollutants and HbA1c are not fully explained, PM_2.5_ and NO_2_ share similar biological mechanisms in the human body: both oxidative and endoplasmic reticulum stress, systemic and visceral adipose tissue inflammation, and endothelial and mitochondrial dysfunction, which contribute to abnormal insulin signaling (Honda et al., 2017; Rajagopalan and Brook, 2012; Sun et al., 2009). We therefore expected to find adverse effects on HbA1c levels associated with both PM_2.5_ or NO_2_ sources vary greatly between geographic regions. In Mexico City, soil erosion, and industry form the main sources of PM_2.5 (_Molina et al., 2019_)_. The chemical makeup of Mexico City’s PM_2.5_ consists of sulfates, nitrates, and carbon (Edgerton et al., 1999). These were previously associated with HbA1c levels, and elevated diabetes and cardiovascular risks (Wang et al., 2024). A future analysis assessing source-specific PM_2.5_ effects can shed more light on these findings.

Our study also provides evidence of temperature effects on glycemic status in a geographic area featuring moderate temperatures with low variability. Our study consistently showed decreases in HbA1c levels associated with hotter temperature exposures. Studies investigating the effects of temperature on glycemic status are mixed, with some showing hotter temperatures associated with worse glycemic status and others highlighting colder temperatures as a risk factor (Khoshhali et al., 2021; Alghamdi et al., 2021; Tien et al., 2016). These differences may be attributed to different climate regions, as well as methodological differences between studies. A previous analysis among mothers of the PROGRESS cohort found temperature increases associated with decreases in HbA1c levels (Makri and Stilianakis, 2008). Mechanistically, the observed inverse relationship between ambient temperature and HbA1c in our study is consistent with documented metabolic responses to repeated exposure to heat, as an adaptive response (Périard et al., 2015). The differences between studies may be related to differences in geography, population characteristics, and behavior (Hamilton et al., 2008; Tseng et al., 2005). Our results suggest that the context of a study might be a critical part in how associations are formed and informed.

Our multi-exposure models found slightly stronger associations for both PM_2.5_ and average temperature when considering all three exposures simultaneously. However, we did not find evidence for substantial confounding or interactions between the exposures. A systematic review reported inconsistent evidence for interactions between air pollution and heat, with stronger PM_2.5_ effects on all-cause mortality and cardiovascular disease observed on hotter days (Anenberg et al., 2020). Variability in findings regarding PM_2.5_ and temperature interactions may depend on factors such as exposure ranges, geographic characteristics, or population behaviors. Analyzing the combined effects of air pollutants and temperature is critical within the context of climate change, which is expected to increase exposure to environmental health risk factors (Kinney, 2018) and potentially alter factors that influence interactions between exposures.

Our study has several notable strengths. Our use of geospatial exposure models at a 1-km^2^ scale significantly minimizes exposure measurement error, allowing us to estimate less biased exposure-response associations. Additionally, we simultaneously assess the effects of multiple environmental exposures to account for the confounding effects of these co-exposures and the potential differences by sex and age. Finally, we utilize data from a longitudinal ongoing cohort with rich covariates to investigate environmental health effects in children in an area with unique geographic characteristics.

Our study has a few limitations. About 5 % of our participants were excluded from the analysis due to missing covariate data, which could introduce bias if the missingness is related to the exposures or the outcome of interest. To address this, we conducted a sensitivity analysis using inverse probability weighting to mitigate potential selection bias, and our findings remained consistent. There is also a notable protective effect with NO2 among children aged 6–7, but it is not clear whether this is due to random chance or missing confounder in that age group. Additionally, as in any similar analysis using geospatial models for exposure assessment, there may be exposure measurement errors. However, given the high temporal and spatial resolution of our models, we expect this error to be minimal.

Although we adjust the models for a rich set of confounders, residual confounding by omitted confounders (e.g., physical activity, diet, fluid intake) may still be present. However, the large E-values suggest that substantial unmeasured confounding would be required to nullify the observed associations with PM_2.5_ and temperature, making such a scenario unlikely. Additionally, our findings may not be generalizable to populations vastly different from the PROGRESS population. However, since study participants were recruited from IMSS, Mexico’s largest healthcare provider, which serves over 55 % of the country’s population, the study population is likely representative of Mexico City. Fourth, the health effects of environmental exposures from ambient sources, such as air pollution and temperature, are generally modest, and our findings are consistent with this pattern. However, due to the widespread nature of these exposures, even minor impairments in glucose regulation caused by ambient factors can result in significant population-level impacts (Rajagopalan and Brook, 2012). As glycemic control is a well-established risk factor for cardiovascular disease, slight variations can lead to clinically meaningful changes in disease risk (Gerstein, 1997). Lastly, the limited sample size may have reduced our ability to detect statistical significance in some results, such as the interaction terms for PM_2.5_ and ambient temperature.

## 5. Conclusions

This study provides valuable insights into the simultaneous associations of air pollution and temperature with glycemic control among children in Mexico City. Our findings demonstrate a consistent association between PM_2.5_ exposure and increased HbA1c levels, aligning with prior research, while no association was observed for NO_2_. Additionally, higher temperatures were associated with reduced HbA1c levels, highlighting the complex relationship between ambient temperature and glycemic status.

The observed age and sex-specific differences in susceptibility underscore the importance of considering developmental stages in assessing environmental health impacts. This study reinforces the need for a holistic approach to studying multiple environmental exposures, particularly in vulnerable populations such as children, to better understand their potential long-term health implications. These findings emphasize the urgency of mitigating air pollution and adapting to climate change to protect children’s health in rapidly urbanizing settings.

## Supplementary Material

1

## Figures and Tables

**Table 1:** Descriptive statistics of the study population (repeated measures).

Variable	Summary statistics

**Children (N)/ HbA1c Test Results (N)**	**503/1196**
Age, Mean (SD)	6.80 (2.07)
Sex, N (%)	
*Female*	612 (51.2)
HbA1c, %, Mean (SD)	5.16 (0.51)
Season (%)	
*November-February*	327 (27.3)
*March-April*	182 (15.2)
*May-October*	687 (57.4)
**Mother’s demographics**	
Ever Smoker, N (%)	
*Yes*	738 (61.7)
Marital Status, N (%)	
*Single/Separated*	222 (18.6)
Education Level, N (%)	
*Middle School or Less/Unknown*	476 (39.8)
*High School/Technical School*	513 (42.9)
*College or Graduate*	207 (17.3)

**Table 2:** The relative change in HbA1c levels, associated with one unit increase in PM_2.5_, NO_2_, and ambient temperature averaged over three months before the visit.

Exposure	Single exposure models	Multi-exposure models	Multi-exposure with IPW

	% Change (95 % CI)		

PM_2.5_, 3-months average	0.264 (0.135, 0.393)*	0.311 (0.159, 0.464)*	0.303 (0.151, 0.457)*
NO_2_, 3-months average	0.061 (−0.036, 0.158)	−0.010 (−0.126, 0.106)	−0.025 (−0.141, 0.091)
Temperature, 3-months average	−0.487 (−0.825, −0.148)*	−0.626 (−0.977, −0.274)*	−0.599 (−0.953, −0.244)*

IPW: inverse probability weights

Table 2 shows the single and multi-exposure models for the associations of HbA1c with PM_2.5_, NO_2_, and ambient temperature. In the single exposure models, each exposure is included in a separate model, with adjustment for the children’s age, season, and year of their clinical visit, and their mothers’ smoking status, marital status, and education level. The multi-exposure model contains all three air pollutants while adjusting for the same set of covariates. Finally, the weighted multi-exposure models include inverse probability weights of inclusion in the cohort to account for potential selection bias due to missing covariate data.

* p-value < 0.05

## Data Availability

The authors do not have permission to share data.

## Appendix A. Supporting information

Supplementary data associated with this article can be found in the online version at doi:10.1016/j.ecoenv.2025.119424.
