# Supplementary material for: Air pollution, temperature, and HbA1c levels among children in Mexico City, Mexico
Source: Ecotoxicol Environ Saf. Author manuscript; Available in PMC 2026 Feb 12. (PMC12895383; doi:10.1016/j.ecoenv.2025.119424)
Supplement: 1 [file NIHMS2127579-supplement-1.docx]

F**igure S1.** The associations between PM_2.5_, NO_2_, and ambient temperature and HbA1c levels among children.


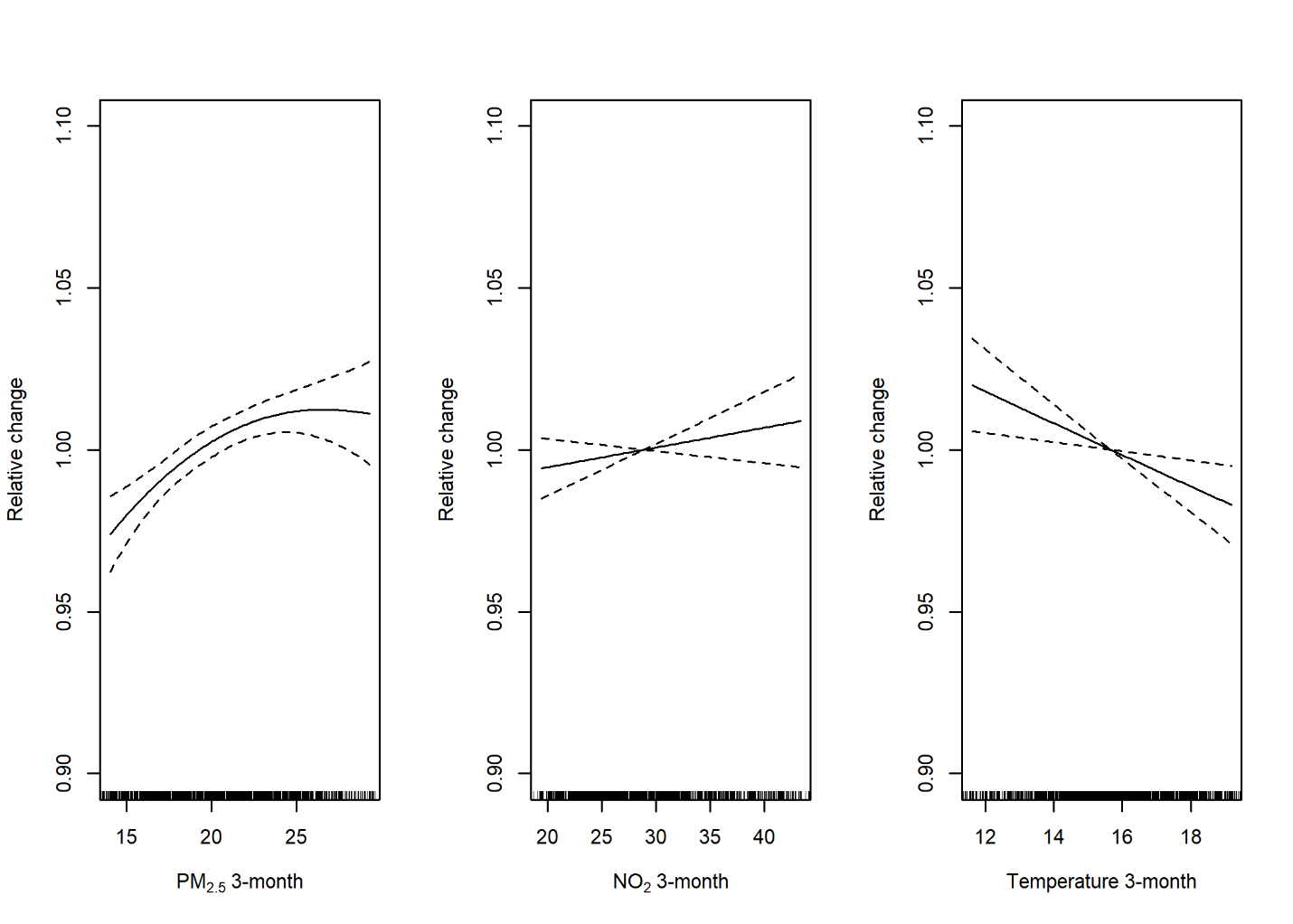


Results were obtained from a multi-exposure model using penalized splines for the exposures and adjusting for the children's age, season, and year of their clinical visit, and their mothers' smoking status, marital status, and education level.

**Figure S2.** Correlation plot of air pollutants and ambient temperature.


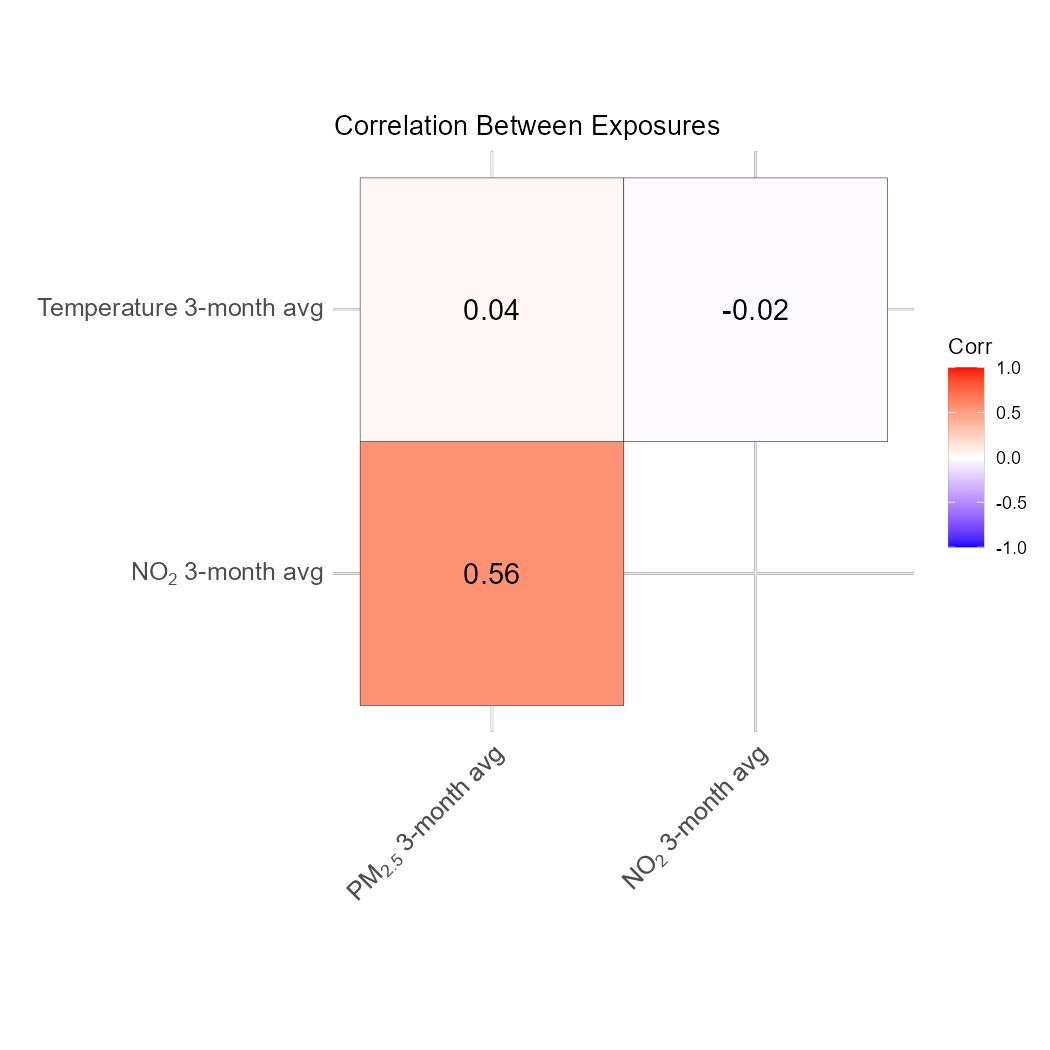


**Table S1.** Descriptive statistics of the study population (repeated measures of child visits) stratified by sex and age group.

| **Variable** | **Summary statistics** | | | | |
| --- | --- | --- | --- | --- | --- |
|  | **Males** | **Females** | **Ages 4 to 5** | **6 to 7** | **8 to 11** |
| **HbA1c Test Results** | 584 | 612 | 336 | 399 | 461 |
| Age, Mean (SD) | 6.81 (2.09) | 6.79 (2.05) | 4.32 (0.47) | 6.20 (0.40) | 9.14 (0.73) |
| Sex, N (%) |  |  |  |  |  |
| *Female* | 0 (0.0) | 612 (100.0) | 169 (50.3) | 210 (52.6) | 233 (50.5) |
| HbA1c, %, Mean (SD) | 5.13 (0.54) | 5.19 (0.48) | 5.19 (0.41) | 5.39 (0.29) | 4.94 (0.62) |
| Season (%) |  |  |  |  |  |
| *November-February* | 171 (29.3) | 156 (25.5) | 91 (27.1) | 125 (31.3) | 111 (24.1) |
| *March-April* | 77 (13.2) | 105 (17.2) | 68 (20.2) | 77 (19.3) | 37 (8.0) |
| *May-October* | 336 (57.5) | 351 (57.4) | 177 (52.7) | 197 (49.4) | 313 (67.9) |
| **Mother’s demographics** |  |  |  |  |  |
| Ever Smoker, N (%) |  |  |  |  |  |
| *Yes* | 339 (58.0) | 399 (65.2) | 218 (64.9) | 257 (64.4) | 263 (57.0) |
| Marital Status, N (%) |  |  |  |  |  |
| *Single/Separated* | 86 (14.7) | 136 (22.2) | 65 (19.3) | 70 (17.5) | 87 (18.9) |
| Education Level, N (%) |  |  |  |  |  |
| *Middle School or Less/Unknown* | 235 (40.2) | 241 (39.4) | 132 (39.3) | 162 (40.6) | 182 (39.5) |
| *High School/Technical School* | 229 (39.2) | 284 (46.4) | 148 (43.8) | 146 (41.4) | 147 (41.6) |
| *College or Graduate* | 120 (20.5) | 87 (14.2) | 57 (16.9) | 60 (17.0) | 57 (16.1) |
| **Exposures** |  |  |  |  |  |
| PM_2.5_ 3month, Mean (SD) | 20.55 (4.27) | 20.88 (4.17) | 21.56 (4.33) | 21.52 (3.95) | 19.42 (4.03) |
| NO_2_ 3month, Mean (SD) | 28.50 (5.81) | 29.23 (6.46) | 30.66 (6.16) | 30.15 (6.11) | 26.47 (5.40) |
| Temperature 3month, Mean (SD) | 15.70 (1.91) | 15.64 (2.03) | 15.22 (1.90) | 15.26 (2.01) | 16.35 (1.80) |

**Table S2:** The associations between PM_2.5_, NO_2_, ambient temperature, and HbA1c levels stratified by sex and age group.

|  | **% Change (95% CI)** | | | | |
| --- | --- | --- | --- | --- | --- |
| **Exposure** | **Sex** | | **Age group** | | |
|  | **Males** (N= 584) | **Females** (N= 612) | **4 & 5** (N= 336) | **6 & 7** (N= 399) | **8 & over** (N= 461) |
| PM_2.5_, 3-months average (95%CI) | 0.366 (0.119, 0.613) * | 0.245 (0.065, 0.426) * | 0.132 (-0.116, 0.382) | 0.325 (0.120, 0.531) * | 0.577 (0.289, 0.866) * |
| NO_2_, 3-months average (95%CI) | -0.095 (-0.291, 0.103) | 0.050 (-0.084, 0.184) | 0.028 (-0.154, 0.211) | -0.265 (-0.401, -0.130) * | 0.190 (-0.035, 0.415) |
| Temperature, 3-months average (95%CI) | -0.480 (-1.050, 0.095) | -0.829 (-1.246, -0.410) * | -0.466 (-1.062, 0.134) | 0.131 (-0.261, 0.526) | -1.396 (-2.062, -0.726) * |

* p-value < 0.05

Multi-pollutant models contain all three exposures in a single model while adjusting for the children's age, season, and year of their clinical visit, as well as their mothers' smoking status, marital status, and education level. Percent changes are calculated for each unit change of exposure.

**Table S3.** The relative change in HbA1c levels, associated with one unit increase in PM_2.5_, NO_2_, and ambient temperature averaged over six months before the visit.

| **Exposure** | **Single pollutant models** | **Multi-pollutant models** | **Multi-pollutant with IPW** |
| --- | --- | --- | --- |
|  | **% Change (95% CI)** | | |
| PM_2.5_, 6-months average | 0.227 (0.026, 0.428) * | 0.289 (0.034, 0.544) * | 0.290 (0.035, 0.545) * |
| NO_2_, 6-months average | 0.017 (-0.082, 0.116) | -0.026 (-0.157, 0.105) | -0.042 (-0.172, 0.089) |
| Temperature, 6-months average | -0.484 (-0.839, -0.128) * | -0.522 (-0.896, -0.146) * | -0.495 (-0.872, -0.117) * |

* p-value < 0.05

IPW: inverse probability weights

In the single exposure models, each exposure is included in a separate model, with adjustment for the children's age, season, and year of their clinical visit, and their mothers' smoking status, marital status, and education level. The multi-exposure model contains all three exposures while adjusting for the same set of covariates.

**Table S4.** The calculated E-values to quantify the effects of possible missing confounders in our models. Estimates were transformed into risk ratios, and reciprocals were used for risk ratios less than 1 to facilitate consistent interpretation of E-values on the risk-increasing scale.

| **Exposure** | **Risk Ratio (95% CI)** | **E-value (RR)** | **E-Value (95% CI)** |
| --- | --- | --- | --- |
| PM_2.5_ | 1.60 (1.27, 2.01) | 2.58 | 1.86 |
| Temperature | 2.38 (1.30, 4.35) | 4.20 | 1.93 |

RR: Risk Ratio, CI: confidence interval.

E-values quantify the minimum strength of association that an unmeasured confounder would need to have with both the exposure and the outcome, on the risk ratio scale, to fully explain away the observed association. The E-value for the point estimate reflects the robustness of the main result, while the E-value for the confidence interval indicates the robustness of the limit closest to the null. Larger E-values suggest greater robustness to unmeasured confounding.
